# Open Foramen Ovale

**Published:** 1833

**Authors:** 


### IV. Open Foramen Ovale.

Dr. J. W. Hall, of Frankfort, Kentucky, has transmitted to us the notes of a case of unclosed foramen ovale.

"The following case of mal-formation of the heart, presented itself to me last summer, in the infant daughter of Mr. M. of this place, which died when six months old. From birth it had blueness of the skin, but in all other respects it seemed to enjoy perfect health. At the age of about two months, it was suddenly attacked with an anxious, struggling difficulty of breathing, amounting almost to suffocation, with an increased lividness of the skin, particularly that of the lips, fingers and toes. The pulse was increased in frequency, and very irregular. The heart labored so greatly, as to agitate the whole body of the child by its action. The paroxysm generally lasted about half an hour, leaving the patient in a languid, exhausted, and placid state. Great care was necessary in the dressing of it, as the least exertion or agitation brought on an attack. It had from one to three of these paroxyms every twenty four hours, for about two months and a half, when I recommended a different nurse.

From this time the paroxysms almost ceased, as it had but two or three for five weeks. From the time of the changing the nurse, however, it declined in flesh, and died in about five weeks.

On examining it after death, the *foramen ovale* was found so open, as to permit the passage of a small quill, from one auricle to the other. No other congenital defect existed. Some parts of the intestinal parietes were thickened."

We saw this little patient several times, while its parents were on a visit to Cincinnati, and feeling desirous of witnessing the effect of the stimulating bath, recommended so strongly by Dr. Hossack, prescribed accordingly. Every attempt however, to immerse the unfortunate little sufferer, brought on a fit of crying; attended with so much dyspnœa, that it was necessary to discontinue the trials, before a fair experiment could be made. Its circulation was languid, and the skin much reduced in excitement. Its alvine discharges were slow and vitiated, calling for the use of cathartics. Some part of the intestinal derangement, arose, perhaps, from the use of laudanum, of which it took considerable quantities, and generally with advantage.

